# Association between Polygenetic Risk Scores of Low Immunity and Interactions between These Scores and Moderate Fat Intake in a Large Cohort

**DOI:** 10.3390/nu13082849

**Published:** 2021-08-19

**Authors:** Sunmin Park, Suna Kang

**Affiliations:** 1 Obesity/Diabetes Research Center, Department of Food and Nutrition, Hoseo University, Asan 31499, Korea; roypower003@naver.com; 2 Department of Bio-Convergence System, Hoseo University, Asan 31499, Korea

**Keywords:** white blood cells, immunity, polygenetic risk scores, metabolic syndrome, vitamin D, cancer

## Abstract

White blood cell (WBC) counts represent overall immunity. However, a few studies have been conducted to explore the genetic impacts of immunity and their interaction with lifestyles. We aimed to identify genetic variants associated with a low-WBC risk and document interactions between polygenetic risk scores (PRS), lifestyle factors, and nutrient intakes that influence low-WBC risk in a large hospital-based cohort. Single nucleotide polymorphisms (SNPs) were selected by genome-wide association study of participants with a low-WBC count (<4 × 10^9^/L, *n* = 4176; low-WBC group) or with a normal WBC count (≥4 × 10^9^/L, *n* = 36,551; control group). The best model for gene-gene interactions was selected by generalized multifactor dimensionality reduction. PRS was generated by summing selected SNP risk alleles of the best genetic model. Adjusted odds ratio (ORs) of the low-WBC group were 1.467 (1.219–1.765) for cancer incidence risk and 0.458 (0.385–0.545) for metabolic syndrome risk. Vitamin D intake, plant-based diet, and regular exercise were positively related to the low-WBC group, but smoking and alcohol intake showed an inverse association. The 7 SNPs included in the best genetic model were *PSMD3*_rs9898547, *LCT*_rs80157389, *HLA-DRB1*_rs532162239 and rs3097649, *HLA-C* rs2308575, *CDKN1A*_rs3176337 and *THRA*_rs7502539. PRS with 7 SNP model were positively associated with the low-WBC risk by 2.123-fold (1.741 to 2.589). PRS interacted with fat intake and regular exercise but not with other nutrient intakes or lifestyles. The proportion with the low WBC in the participants with high-PRS was lower among those with moderate-fat intake and regular exercise than those with low-fat intake and no exercise. In conclusion, adults with high-PRS had a higher risk of a low WBC count, and they needed to be advised to have moderate fat intake (20–25 energy percent) and regular exercise.

## 1. Introduction

The immune system is composed of innate and adaptive immunity systems. When foreign materials and pathogens enter the body, innate immunity is activated, and adaptive immunity is subsequently initiated. Immunity dysregulation results in immune deficiency or immune system overactivation [1]. As encountered in lymphopenia, immune exhaustion renders the individual susceptible to infection, certain cancers and sepsis, whereas its overactivation is associated with autoimmune diseases. White blood cells (WBC), also called leukocytes, are responsible for innate and adaptive immunity [2]. WBCs protect the body against bacterial and viral infections and are more strongly linked to the innate immune system [3]. Natural killer cells, mast cells, macrophages, eosinophils, basophils, neutrophils, and dendritic cells are components of the innate immune system, whereas other less numerous WBC, including B and T cells, are components of the adaptive immune system [4]. Therefore, WBC is associated mainly with innate immunity to fight against pathogen attacks, and it is involved partly in adaptive immunity [4].

Immediate response to a noxious challenge is achieved by activating the innate immune system, which manifests as the rapid induction of acute inflammation. However, low immunity fails to fight against pathogens susceptible to severe infection and potential cancer risk, while subclinical immune overactivation induces persistent inflammation, called chronic low-grade inflammation [5,6]. Persons with either low- or overactive immunity are susceptible to infection severity and mortality during pathogen attack since persons with low immunity cannot efficiently eliminate pathogens and those with overactive immunity have a high chance of inducing cytokine storms [1]. Thus, optimal regulation of immunity is a strategy used to reduce metabolic syndrome (MetS), infection, and cancer risk [3].

No promising biomarkers of immunity are available in clinical settings, but WBC counts are generally used to assess the immune status [7]. WBC counts provide an easily accessed and reliable biomarker of overall immunity in the clinical setting. Under normal circumstances, WBC counts are considered to range from 4 to 11 × 10^9^/L. However, a WBC count of 6.2 × 10^9^/L is determined as the cutoff point for elevated metabolic syndrome risk in the Korean population [2]. Therefore, a WBC count of 4–6.2 × 10^9^/L is considered normal in a narrow definition. High WBC counts are related to cigarette smoking, splenectomy, bacterial infection, inflammatory disease, leukemia, and tissue damage. In contrast, low WBC counts are associated with bone marrow deficiency or failure, liver or spleen diseases, viral infections, cancers, cancer medication, and severe emotional or physical stress. Therefore, low and high WBC counts are involved in the etiology of different diseases.

WBC counts are linked to genetic predisposition and lifestyle factors and their interactions. At the genetic level, WBC counts are associated with human leukocyte antigen (*HLA*)*-C*, *HLA-G*, *HLA-DQA1/DRB1*, interleukin (*IL)-10*, and cluster of differentiation 4 (*CD4*) polymorphisms, which are known to be involved in various immune-related diseases [8,9,10,11]. Genetic variants of these polymorphisms have been mainly studied in the context of immunodeficiency [8,12,13]. Individuals with the *HLA-C* rs9264942 TT genotype demonstrate significantly higher human immunodeficiency virus type 1 (HIV-1) viral loads than those with the CC genotype [8]. The rs1518111 and rs1800872 polymorphisms of *IL-10* (a potent anti-inflammatory cytokine) are associated with low *CD4* T-cell counts in HIV patients [13]. *HLA-B* polymorphism has been reported to be associated with penicillin allergy [12], and the *HLA-DQA1/DRB1* polymorphism is found to be significantly associated with hepatocellular carcinoma development (hazard ratio 4.91, 95% CI = 1.41–17.11, *p* = 0.01) [10]. WBC counts also interact with lifestyles, but only a few studies have addressed their interactions. We aimed to identify the genetic variants associated with low-WBC counts (<4 × 10^9^) and the interaction of their polygenetic risk scores (PRS) with lifestyles, including nutrient intake, smoking, and physical exercise in a large hospital-based cohort. In addition, we examined the relation between PRS and cancers and metabolic syndrome risk.

## 2. Methods

### 2.1. Participants

Korean adults >40 years old (*n* = 58,645) were voluntarily recruited to participate in the hospital-based urban cohort of Korean Genome and Epidemiology Study (KoGES) organized by the Korean Center for Disease and Control during 2004–2013. The replicate study for determining obesity-related genetic variants was conducted in 5493 adults aged 40–79 years to have Korean Chip data in the Ansan/Ansung cohort. The institutional review board of the Korean National Institute of Health approved KoGES (KBP-2015-055), and the protocol of the present study was approved by the institutional review board Hoseo University (1041231-150811-HR-034-01). Written informed consent was obtained from all participants.

### 2.2. Anthropometric and Biochemical Measurements

Information on age, gender, residence area over at least the previous six months, educational level, income, smoking status, physical activities, and daily alcohol consumption were obtained during a health interview [14]. When the participants conducted moderate-intensity exercise for 30 min more than three times a week (>150 min/week), they were considered as having regular exercise (physical activity). Moderate-intensity exercise included fast walking, mowing, badminton, swimming, tennis, and jogging. The participants were divided into two groups with and without regular exercise. Smoking status was categorized as current smoker, past smoker, or never-smoker [15] and alcohol consumption as nondrinker (0 g/day), mild drinker (0–20 g/day), and moderate drinker (>20 g/day) [15].

Trained technicians measured body weights, heights, and waist circumferences using standard procedures [16]. Body mass index (BMI) was calculated by dividing weight (kg) by height (m) squared. Blood was drawn after a ≥12 h fast (no food or water), and plasma and serum were separated for biochemical analysis [16]. Fasting plasma glucose concentrations and serum lipid profiles were measured using a Hitachi 7600 Automatic Analyzer (Hitachi, Tokyo, Japan). Serum high-sensitive C-reactive protein (hs-CRP) concentrations were measured by ELISA kit. WBC counts were conducted using EDTA-treated blood. Blood pressures were measured on right arms at heart height in a sitting position after resting for over 10 min.

### 2.3. Definition of Immunity and MetS

The participants were divided into two groups (<4 × 10^9^/L and ≥4 × 10^9^/L) according to WBC counts for genetic analysis of the low-WBC risk. They were also categorized into three immunity groups, including <4 × 10^9^/L, 4–6.2 × 10^9^/L, and ≥6.2 × 10^9^/L for metabolic analysis. The participants with MetS were categorized according to the 2005 revised National Cholesterol Education Program-Adult Treatment Panel III criteria for Asia as described in the previous studies [17,18]. The participants answered the history of immunity-related diseases, including allergy, gastritis, asthma, bronchitis, and arthritis, used as confounding variables.

### 2.4. Dietary Pattern Analysis from Semi-Quantitative Food Frequency Questionnaire (SQFFQ)

Usual food intakes were determined using an SQFFQ developed and validated for KoGES [19]. The questionnaire included 106 food items, and participants selected item frequencies and serving sizes (from among 1/2, 1, or 2 serving sizes). Overall consumptions were calculated by multiplying item frequencies by serving sizes, and nutrient intakes were calculated from daily food intakes using a Computer-Aided Nutritional Analysis Program (ver. 3.0) developed by the Korean Nutrition Society [19].

The 106 food items were categorized into 29 food types used as independent variables in the factor analysis to determine dietary patterns. The number of factors retained after principal component analysis (PCA) was determined using eigenvalues of >1.5 and the orthogonal rotation procedure (Varimax) [20]. Food groups with factor loadings ≥0.40 significantly contribute to assigning dietary patterns. Four distinct dietary patterns, the Korean balanced diet (KBD), plant-based diet (PBD), Western-style diet (WSD), and rice-main diet (RMD), were selected for Korean dietary patterns. The factor loadings of food groups in the four dietary patterns were presented in Supplemental Table S1.

### 2.5. Dietary Inflammatory Index (DII)

DII was calculated from individual food and nutrient intakes having the potential for anti-inflammation using their dietary inflammatory weights for certain foods and nutrients (energy, 32 nutrients, four food products, four spices, and caffeine), as previously described [21]. It indicated the anti-inflammatory food intake of the participants. Since the SQFFQ did not include spice intakes, we excluded garlic, ginger, saffron, and turmeric intake from DII calculations. DIIs were calculated by multiplying the dietary inflammatory weights of the 38 food and nutrient components by daily intakes and dividing the sum of these products of 38 items by 100.

### 2.6. Quality Control of Genotyping

Individual genetic variants were determined by the Center for Genome Science at the Korea National Institute of Health and provided for further study. Genomic DNA was extracted from whole blood, and genetic variants were determined using a Korean Chip (Affymetrix, Santa Clara, CA, USA) designed for Korean genetic research that included known disease-related SNPs [22]. Genotyping accuracy was confirmed with Bayesian robust linear modeling with the Mahalanobis Distance Genotyping Algorithm [23]. In quality control, genetic variants were selected with dish quality control (>0.82) and call rates (>98%) and excluded low-quality SNPs by Axiom Analysis Suit Guideline from ThermoFisher (Waltham, MA, USA). All genetic variants satisfied Hardy-Weinberg equilibrium (HWE) inclusion criteria (*p* > 0.05), and the genotype missing rate was less than 5% [22].

### 2.7. Genetic Variants for Low-WBC Count Risk

**Figure 1:**
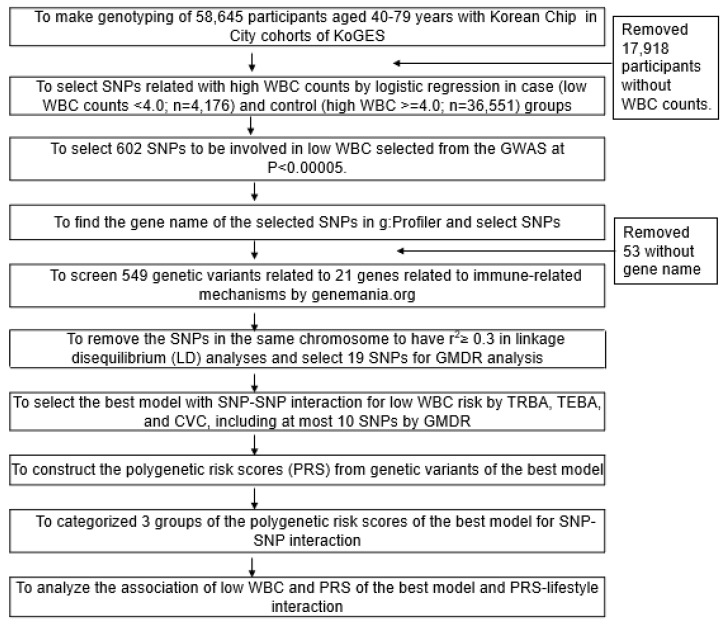
Flow chart for the generation of polygenetic variants increasing the risk of a low white blood cell (WBC) count and interactions between polygenetic risk scores (PRS) and lifestyles.

The flow chart used to generate polygenetic risk scores (PRS) for low WBC count (<4 × 10^9^/L) risk is shown in Figure 1. Participants were divided into low-WBC group (*n* = 4176) or a control (*n* = 36,551) group in the hospital-based cohort. GWAS was conducted to explore genetic variants associated with low-WBC risk at *p* < 0.00001 to have a big pool of SNPs to generate the best model explaining immunity-related pathways using PLINK 2.0 (http://pngu.mgh.harvard.edu/~purcell/plink (accessed on 12 January 2021)). The 602 genetic variants were selected from the GWAS, and their gene names were identified using g:Profiler (https://biit.cs.ut.ee/gprofiler/snpense (accessed on 26 January 2021)). Fifty-three genetic variants without corresponding gene names were excluded. Genes of the 549 SNPs selected were screened for immunity, and 21 genes were corresponding to 549 SNPs. The SNPs were then checked for linkage disequilibrium (LD) in the same chromosomes using LocusZoom (https://genome.sph.umich.edu/wiki/LocusZoom_Standalone (accessed on 3 February 2021), and those with weak LD (r^2^ < 0.3) were included. Nineteen SNPs were left from LD analysis, and their genetic characteristics were shown in Supplemental Table S2.

As the replicate study, the adjusted ORs of the selected SNPs for the best model were analyzed for low-WBC risk in the 5493 participants in Ansan/Ansung cohort who determined genetic variants with Korean Chip. The number of participants in the case and control groups was 207 and 5286, respectively.

### 2.8. The Best Model for Gene–Gene Interactions of Genetic Variants by Generalized Multifactor Dimensionality Reduction (GMDR)

The 19 SNPs were used to find the best model using GMDR, and the final best model included 10 SNPs. The best model was chosen with the interactions of potential genetic variants for the low-WBC count by GMDR [17]. Using GMDR, the best SNP-SNP interaction model was selected using a *p*-value of <0.05 by the sign rank test with trained balanced accuracy (TRBA) and testing balanced accuracy (TEBA) with adjustment for the covariates of age and gender, living area, body mass, and serum hs-CRP concentration [24]. Ten-fold cross-validation was used to check cross-validation consistency (CVC) since the sample size was larger than 1000 [24]. The risk and non-risk alleles of each SNP were counted as 1 [25]. For example, when the C allele was associated with an increased risk of the low-WBC count, TT, CT, and CC were assigned 0, 1, and 2. PRS was obtained by summing the number of risk alleles in the best model. PRS of the best model containing 2 or 7 SNPs were categorized as (0–1, 2, and ≥3) and (0–5, 6–7, and ≥8) by PRS, referred to as low-, medium-, and high-PRS groups. A high-PRS indicated a higher number of risk alleles in the best genetic variant-genetic variant interaction model.

### 2.9. Statistical Analyses

Statistical analysis was conducted using SAS version 9.3 (SAS Institute, Cary, NC, USA). Descriptive statistics for categorical variables (e.g., gender and lifestyle) were calculated based on frequency distributions according to WBC and PRS groups. Frequency distributions of categorical variables were analyzed using the chi-squared test. WBC counts were classified as <4.0 × 10^9^, 4–6.2 × 10^9^, and ≥6.2 × 10^9^/L to determine the effects of WBC counts on metabolic syndrome and its components. Adjusted means and standard errors were determined for continuous variables of the control and low-WBC group. The significant differences between the low-WBC and control groups were determined by analyzing covariance (ANCOVA) with covariate adjustment. After covariate adjustment, adjusted odds ratios (ORs) and 95% confidence intervals (CI) of metabolic syndrome and its components for low-WBC risk were calculated by multiple logistic regression analysis.

Adjusted ORs and 95% confidence intervals (CIs) of PRS for low-WBC risk or MetS and its components were analyzed after adjusting for covariates. According to the different covariates, two models were included for PRS for low-WBC risk: model 1 included age, gender, residence area, survey year, income, and education level as covariate and model 2 contained the variables in model 1 plus energy intake, smoking status, physical activity, alcohol intake, autoimmune diseases, and serum hs-CRP concentrations as variates. In other logistic regression analyses, covariates in model 2 were used. Participants were categorized into high and low dietary intake groups to examine the interactions between PRS and dietary intake parameters. Two-way ANCOVA with main effects and an interaction term were used to investigate interactions between PRS and lifestyle parameters that affect low-WBC risk after adjusting for covariates. Statistical significance was accepted for *p* values < 0.05.

## 3. Results

### 3.1. General Characteristics of Participants in the WBC Count Groups

The participants in the low-WBC group were older than those in the control group. In the low-WBC group, men were much lower than women. Adjusted ORs for genders were inversely associated with WBC counts after adjusting for MetS-related parameters, indicating men were inversely associated with the low-WBC risk (Table 1). Mean serum hs-CRP concentration was higher in the middle-WBC and high-WBC group than in the low-WBC group, and serum hs-CRP concentration was inversely associated with WBC counts by 0.542-fold. Cancer incidence was higher in the low-WBC group than in the other groups, and adjusted ORs were positively associated with WBC count by 1.467-fold (cutoff: <4.0) (Table 1). The prevalence of MetS was much higher in the low-WBC group than in the other groups, and the components of MetS, including waist circumferences, plasma glucose, total cholesterol, LDL, and TG, concentrations, SBP, and DBP, showed the same trends. MetS was inversely associated with a low-WBC count (<4.0 × 10^9^/L) by 0.458-fold. Mean waist circumference was higher in the control group than in the low-WBC group, and hip circumference was inversely associated with WBC by 0.86-fold (Table 1).

### 3.2. Lifestyles and Nutrient Intakes

Proportions of smokers and former smokers were much lower in the low-WBC group than in the other groups, and smokers and former-smokers were inversely related to low-WBC risk by 0.298- and 0.352-folds on the reference of the non-smokers (Table 2). However, the proportion of individuals that exercised regularly was higher in the low-WBC group than in the other groups, and regular exercise was positively associated with low-WBC risk by 1.262-fold. There was a higher proportion in the low-WBC group in low alcohol and coffee intake than the other groups (Table 2). Alcohol and coffee intakes were inversely associated with low-WBC risk by 0.849- and 0.856-fold, respectively. Participants in the low-WBC group had lower energy intake and fat intakes than those in the high-WBC group. However, protein intakes were non-significantly different. Interestingly, vitamin D intakes were significantly higher in the low-WBC group than in the high-WBC group (Table 2), and inflammation indices were not significantly different.

### 3.3. Genetic Variants Associated with Low-WBC Count Risk and Gene–Gene Interactions between Genetic Variants by GMDR

Genetic variants associated with low-WBC risk were selected from GWAS results, and ten genetic variants related to immunity and inflammation were selected. Table 3 listed ten selected genetic variants. Ten selected variants were located in chromosomes 2, 6, 7, 17, and 19, while six were located in 6p21 loci, and their LD was r^2^ < 0.3. Genetic variant–variant interactions were investigated using GMDR (Table 3). All selected SNPs satisfied HWE (*p* > 0.05) and MAF (>0.05) criteria (Table 3). In Ansan/Ansung cohort, the selected 10 SNPs exhibited similar OR values to those in a hospital-based cohort, but the significance levels were higher in Ansan/Ansung cohort than those in the hospital-based cohort (Table 3) since the number of cases (*n* = 204), and control (*n* = 5286) was much smaller in Ansan/Ansung cohort.

**Figure 2:**
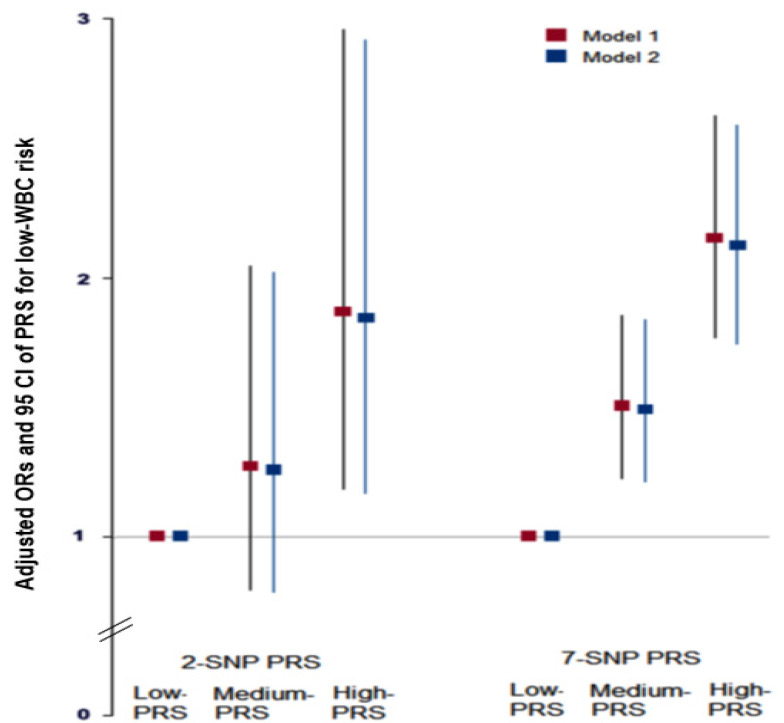
Adjusted odds ratios and 95% confidence intervals of the PRS of 2- and 7-SNP models generated from assessing gene-gene interactions associated with a low white blood cell (WBC) count. PRS of the 2- and 7-SNPs were calculated by summing the number of risk alleles of SNPs. PRS calculated using the 2- and 7-SNPs models were divided into three categories (0–1, 2, and ≥3) or (0–5, 6–7, and ≥8), respectively. Adjusted ORs were obtained by logistic regression after adjusting for various covariates. Two models were composed of different covariates: Model 1 included age, gender, residence area, survey year, income, and education level as covariates, and model 2 contained the variables in model 1 plus energy intake, smoking status, physical activity, alcohol intake, autoimmune diseases, and serum high-sensitive C-reactive protein concentrations as variates. The low-PRS group was used as a reference for logistic regression. Based on the covariates, red and blue boxes indicated adjusted ORs for models 1 and 2, respectively, and lines indicated 95% CIs.

Gene–gene interaction models with 2, 7, 8, 9, or 10 SNPs met the best model criteria. Among them, adjusted ORs of the 2 and 7 SNP models increased low-WBC risk (<4.0 × 10^9^) by 1.844 (1.165–2.918) and 2.123 (1.741–2.589) folds in participants with high-PRS than those with low-PRS (Figure 2). The best model with 2 SNPs included proteasome 26S Subunit, non-ATPase 3 *(PSMD3)*_rs9898547 and lactase (*LCT*)_rs80157389, while that with 7 SNPs contained the SNPs in the 2 SNP model, *HLA-DRB1*_rs532162239 and rs3097649, *HLA-C*_rs2308575, cyclin-dependent kinase inhibitor 1A (CDKN1A)_rs3176337, and thyroid hormone receptor alpha (*THRA*)_rs7502539 (Table 4). Although the 2 SNPs model sufficiently showed increases in the risk of a low-WBC count, we considered that 2 SNPs might be too small to show associations with metabolic syndrome. Additionally, variation was much greater in the 2 SNP model than in the 7 SNP model. Therefore, we used the 7 SNP model to investigate interactions with lifestyles.

### 3.4. Associations between PRS Derived from the 7-SNP Model and MetS and Its Components

There was no significant association between PRS and MetS after adjusting for covariates (Supplementary Table S3). BMI and body fat mass also did not have an association with PRS (Supplementary Table S3). MetS components including waist circumferences, plasma glucose, HDL, triglyceride concentrations were not associated with PRS after adjusting for covariates (Supplementary Table S3). Serum hs-CRP concentrations did not have any relation with PRS (Supplementary Table S3).

### 3.5. Interaction between PRS and Nutrient Intakes and a Low WBC Count Risk

**Figure 3:**
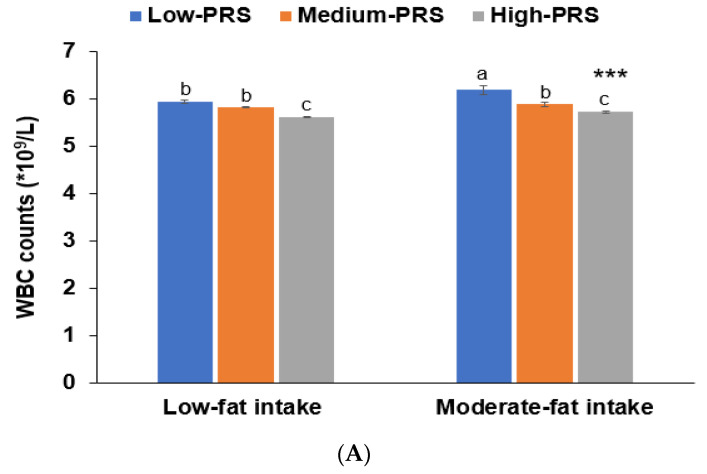

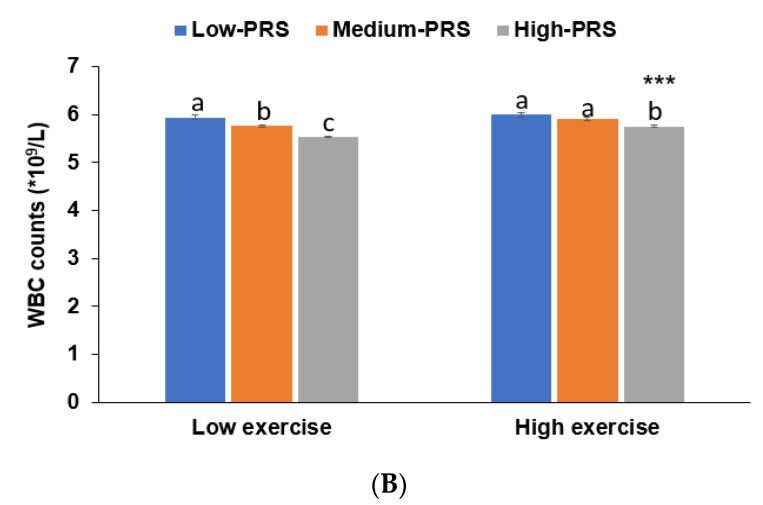
Adjusted means and standard errors of white blood cell counts among 7-SNP polygenetic risk scores (PRS) according to low and high groups of fat intake and exercise. (**A**). Adjusted means and standard errors of WBC in the participants were categorized by fat intake (cutoff value: 20 energy % of fat intake). (**B**). Adjusted means and standard errors of participants categorized by exercise (cutoff: moderate-intensity exercise at least for 30 min three times weekly). *** Significantly different between those with low and high-PRS in ANCOVA at *p* < 0.001. ^a,b,c^ Different alphabetical letters on the bar indicate significant differences among the groups in Tukey’s test at *p* < 0.05.

No interactions were observed between PRS and age, gender, BMI, or metabolic syndrome that affected low-WBC count risk (*p* > 0.05). There was no interaction between lifestyles (except fat intake), PRS, and low-WBC risk (*p* = 0.008; Table 5). In participants with a high fat intake, those with a low-PRS had much higher WBC counts than those with a medium or high-PRS (Figure 3). This trend was similar in the low-fat intake. The association between PRS and WBC counts was greater for participants with high-fat intakes than low-fat intakes (Table 5).

## 4. Discussion

Innate and adaptive immunity is critical in eliminating coronaviruses from the body, but an uncontrolled immune response induces cytokine storms that aggravate acute lung injury. Genetic and environmental factors influence immunity. However, the biomarkers of immunity are difficult to define. WBC can be a biomarker for immunity. The present study demonstrated that the best genetic model for immunity representing WBC counts and the genetic impact interacted with nutrient intake. The best model with 7 SNP model included *PSMD3*_rs9898547, *LCT*_rs80157389, *HLA-DRB1*_rs532162239 and rs3097649, *HLA-C* rs2308575, *CDKN1A*_ rs3176337, and *THRA*_rs7502539. WBC counts should be maintained within the 4–6.2 × 10^9^/L range due to their relationships with cancer and MetS risk, and individuals with a high-PRS for a low-WBC count should be advised to moderate fat intake to 20–25% of total energy consumption. This study is the first to show the interaction of genetic impact and lifestyles that can be applied to personalized nutrition.

At the beginning of infection, immune response by WBCs is critical. In a Mendelian randomization study, WBC count (OR = 0.84, 95% CI = 0.72–0.98), myeloid WBC count (OR = 0.81, 95% CI = 0.70–0.94), granulocyte count (OR = 0.84, 95% CI = 0.71–0.99), and basophil count (OR = 0.75, 95% CI = 0.59–0.96) were found to exhibit inverse associations with severe COVID 19 [26], suggesting that a low-WBC count is associated with increased risk of severe infectious diseases. However, previous studies have reported that circulating WBC counts, including leukocytes, neutrophils, lymphocytes, basophils, and monocytes, are positively associated with MetS, BMI, hypertension, and serum triglyceride concentrations but not glycemic index or insulin resistance [27]. However, in the present study, WBC counts were positively associated with serum glucose concentrations and HbA1c contents. As regards WBC subtypes, blood eosinophil counts were reported to be positively associated with MetS (OR 1.41) and obesity (OR 1.16) [28]. Lymphocytes and neutrophils are also associated with MetS risk [29]. Increases in WBC counts may be related to enhancing antioxidant defense to remove oxidative products [26]. In the present study, a low-WBC count (<4 × 10^9^/L) was positively associated with cancer risk but inversely with MetS risk. Since the cutoff for a low WBC count is 4.0 × 10^9^/L, and that of MetS risk was found to be 6.2 × 10^9^/L in our previous study, we suggest WBC counts better be maintained range 4 to 6.2 × 10^9^/L to reduce the risks of cancer and MetS.

WBC contents are related to disease and nutrition statuses, but genetic predispositions also influence them. The best models generated by genetic variant-genetic variant interactions included *PSMD3*_rs9898547, *LCT*_rs80157389, *HLA-DRB1*_rs532162239 and rs3097649, *HLA-C* rs2308575, *CDKN1A*_rs3176337, and *THRA*_rs7502539. These selected genetic variants arose in genes associated with immunity (*HLA-DRB1, HLA-C,* and *PSMD3)*, energy metabolism (*THRA*), and other functions (*LCT* and *CDKN1A*). MHC is a large gene complex containing over 200 genes located on chromosome 6 at 6p21.3 and plays a vital role in immunity [30]. The selected genes included *HLA-C*, *DRB1*, *DPA1,* and *DPB1*. *HLA* is a human MHC-encoded glycoprotein and encodes HLA-peptide-T cell receptor, which induces adaptive immunity, and *HLA* and *HLA-DRB1* genetic variants reduce viral infection susceptibility [31]. A recent study reported that HLA-B*15:03 has the greatest capacity to present SARS-CoV-2 peptides to immune cells [32], whereas *HLA* is known to be associated with inflammatory, autoimmune, and malignant disorders. Furthermore, *PSMD3 at* 17q21 has been reported to be associated with WBC count in African Americans, Europeans, and Japanese [33]. *PSMD3* promotes nuclear factor kappa-light-chain-enhancer of activated B cells protein expression in chronic myeloid leukemia cells and, thus, promotes disease progression to a chronic state [34]. Therefore, the genetic variants selected for low-WBC risk in the current study.

Interestingly, genetic variants related to *THRA* and *LCT* were associated with low-WBC risk in the present study. Thyroid hormone plays a critical role in thermoregulation and energy expenditure through *THRA* and *THRB* [35]. *THRA* is known to be related to erythropoiesis [36], and its mutation displays the characteristics of hypothyroidism, anemia, and abnormal bone growth [36]. On the other hand, hyperthyroidism has been associated with low WBC counts [37]. *THRA* polymorphisms may influence energy metabolism and WBC production by modulating thyroid hormone activity [38]. *LCT* is an enzyme that converts lactose to glucose and galactose, and *LCT* deficiency induces lactose intolerance and is possibly involved in allergy by modulating immunity. Furthermore, *LCT* polymorphisms are associated with WBC regulation and genetic disposition to CD14 expression, a marker of monocytes and macrophages [39]. In the present study, PRS of the 7-SNP model were positively associated with WBC count by 2.9-fold, but no association was found between PRS and MetS or its components. Asians, including Koreans, are at risk of MetS despite being less obese than Caucasians [40], and, thus, genetic predisposition may explain MetS susceptibility in Korean adults.

Immunity has been reported to be associated with lifestyles. The present study indicates lifestyles could modulate immunity, as reflected by WBC counts: low-WBC counts were inversely associated with current smoking (OR = 0.298), alcohol drinking (OR = 0.849), and coffee drinking (OR = 0.856), but positively associated with regular exercise (OR = 1.262) and a PBD (OR = 1.231) in Korean adults. These results suggest that immunity may be promoted to remove oxidative and pro-inflammatory products elevated in MetS. Immunity, inflammation, oxidative stress, and MetS are known to compose an inter-related vicious cycle, and, thus, reductions in oxidative stress may lower WBC counts. Many studies have reported an association between MetS and lifestyles [41], but not between MetS and WBC counts. The present study showed that the associations between WBC count and lifestyles are similar to those between MetS and lifestyles. However, the impact of the interaction between lifestyles and PRS on low-WBC counts was not significant. Only fat intake interacted with PRS to influence low-WBC counts. In adults, a 20–25% fat intake was optimal in reducing the risks of low-WBC count and MetS.

The present study is the first study to show an association between PRS and low-WBC count and the interaction of PRS with fat intake to modulate WBC counts. However, the present study has several limitations that should be mentioned. First, the study was conducted using a case-control design in a large city hospital cohort study, and data were collected cross-sectionally, which prevented our accessing cause-and-effect relationships. Second, usual intakes of nutrients and foods may have been underestimated or overestimated since they were obtained from SQFFQ results of the consumption of 106 common Korean foods. However, the SQFFQ has been shown to estimate usual intakes reliably and validated using 3-day food records [40]. Third, WBCs were not classified by cellular subtypes, and subtypes may be differentially associated with PRS and MetS. Fourth, the present study did not show the direct effect of WBC counts on immunity since the incidence of cold or virus infection, immunity-related diseases, was not provided in the hospital-based cohort study. However, low WBC counts had an inverse relation with cancer risk in the present study. Recent studies have demonstrated that WBC counts are inversely correlated with fever and severity from coronavirus disease 2019 (COVID-19) infection in the chest damage showing by imaging analysis, including ground-glass opacities and multiple patchy shadows [42,43].

In conclusion, low WBC counts exhibited a positive association with cancer risk and an inverse relation with MetS risk. The best genetic model for low WBC count included the genes related to immunity. Participants with a high-PRS of the best genetic model for low-WBC had a 2.123-fold higher risk of a low WBC count. Furthermore, PRS and fat intake interacted to modulate the risk of low-WBC. Thus, we recommend that Korean adults with low-PRS adopt diets containing 20–25% fat to maintain WBC counts in the range 4–6.2 × 10^9^/L, where the risk of cancer and severity from infectious diseases including COVID-19 might be minimized. This study is the first study to show the interaction of genetic impact and lifestyles to influence a low-WBC risk, and the results can be applied to personalized nutrition. Further study is needed to investigate the effects of high PRS for low WBC and infectious diseases.

## Figures and Tables

**Table 1:** Demographic, anthropometric, and biochemical characteristics of the participants according to the contents of white blood cells (WBC) and their adjusted odds ratio (ORs) of low-WBC and 95% confidence intervals (CI).

	Low (<4) (*n* = 4176)	Normal (4≤ <6.2) (*n* = 23,911)	High (<6.2) (*n* = 12,640)	Adjusted ORs (95% CI) of Low-WBC ^1^^6^
Age ^1^ (years)	54.1 (53.9–54.3) ^1^^4,b^	53.9 (53.8–54.0) ^b^	53.2 (53.1–53.3) ^a,^***	1.134 (1.040–1.237)
Gender (N, male %)	742 (17.8) ^1^^5^	7432 (31.1)	5682 (44.9) ^+++^	0.614 (0.534–0.706)
Cancer (N, Yes %)	283 (6.8)	981 (4.1)	423 (3.4) ^+++^	1.467 (1.219–1.765)
Serum hs-CRP ^2^ (ng/mL)	0.097 (0.083–0.113) ^c^	0.111 (0.105–0.117) ^b^	0.209 (0.201–0.217) ^a,^***	0.542 (0.416–0.706)
Metabolic syndrome (N, Yes %)	239 (5.7)	2860 (12.0)	2625 (20.7) ^+++^	0.458 (0.385–0.545)
BMI ^3^ (kg/m^2^)	23.0 (22.9–23.1) ^c^	23.8 (23.7–23.8) ^b^	24.3 (24.3–24.4) ^a,^***	0.561 (0.510–0.618)
Fat mass ^4^ (%)	1749 (41.9)	11848 (49.5)	6835(53.9) ***	0.541 (0.498–0.588)
Waist circumference ^5^ (cm)	79.8 (79.6–79.9) ^c^	80.3 (80.3–80.4) ^b^	80.8 (80.7–80.9) ^a,^***	0.548 (0.485–0.618)
Plasma glucose ^6^ (mg/dL)	92.7 (92.1–93.3) ^c^	94.7 (94.4–94.9) ^b^	97.3 (97.0–97.7) ^a,^***	0.464 (0.386–0.557)
HbA1c ^7^ (%)	5.56 (5.58–5.59) ^c^	5.67 (5.66–5.68) ^b^	5.85 (5.84–5.87) ^a,^***	0.376 (0.299–0.473)
Serum total cholesterol ^8^ (mg/dL)	193 (192–194) ^c^	198 (197–198) ^b^	199 (199–200) ^a,^***	0.670 (0.606–0.740
Serum HDL ^9^ (mg/dL)	56.9 (56.5–57.3) ^a^	54.8 (54.6–54.9) ^b^	53.3 (53.1–53.5) ^c,^***	0.725 (0.658–0.800)
Serum LDL ^10^ (mg/dL)	115 (114–116) ^b^	119 (118–119) ^a^	118 (117–119) ^a,^***	0.662 (0.588–0.744)
Serum TG ^11^ (mg/dL)	106 (103–109) ^c^	122 (121–123) ^b^	140 (139–142) ^a,^***	0.516 (0.464–0.574)
SBP (mmHg) ^12^	120.8 (120.4–121.3) ^c^	122.4 (122.2–122.6) ^b^	123.7 (123.4–123.9) ^a,^***	0.817 (0.746–0.895)
DBP (mmHg) ^13^	74.3 (74.0–74.6) ^c^	75.3 (75.1–75.4) ^b^	76.0 (75.8–76.1) ^a,^***	0.799 (0.674–0.946)

The cutoff points were as following: ^1^ <55 years old; ^2^ <0.5 mg/dL for high-sensitive C-reactive protein (hs-CRP), ^3^ <25 mg/kg^2^ for body mass index (BMI), ^4^ <25% for men and <30% for women; ^5^ <90 cm for men <85 cm for women; ^6^ <126 mg/dL fasting serum glucose and ^7^ <6.5% HbA1c or taking hypoglycemic medication; ^8^ <230 mg/dL serum total cholesterol, ^9^ ≤40 mg/dL for men and ≤50 mg/dL serum HDL; ^10^ <160 mg/dL serum LDL, ^11^ <150 mg/dL serum triglyceride; ^12^ <130 mmHg SBP and ^13^ <90 mmHg DBP or taking hypotensive medication.^1^^4^ Values represent adjusted means and 95% confidence intervals (CI) after adjusting for covariates or ^1^^5^ the number of the subjects and percentage. Covariates used were age, sex, body mass index (BMI), energy intake, income, education, residence area, survey year, and autoimmunity-related diseases, including atopic dermatitis, asthma, allergy, and inflammation-related diseases, alcohol intake, smoking status, and physical activity. ^1^^6^ Adjusted odds ratio (ORs) and 95% confidence intervals of each parameter for the hypo-WBC (<4.0 × 10^9^/L) risk after adjusting for covariates in logistic regression analysis. ^a,b,c^ Different letters indicate significant differences among the groups in the Tukey test at *p* < 0.05. *** Significantly different for WBC count groups by one-way ANCOVA in continuous variables at *p* < 0.001. ^+++^ Significantly different WBC count groups by χ^2^ test at *p* < 0.001. hs-CRP, high-sensitive C-reactive protein; HbA1c, blood hemoglobin A1c; LDL, low-density lipoprotein; HDL, high-density lipoprotein; TG, triglyceride; SBP, systolic blood pressure; DBP, diastolic blood pressure.

**Table 2:** Lifestyles and nutrient intake of the participants according to the contents of white blood cells (WBC) and adjusted odds ratio (ORs) of low-WBC.

KERRYPNX	Low (<4) (*n* = 4176)	Normal (4≤ <6.2) (*n* = 23,911)	High (<6.2) (*n* = 12,640)	Adjusted ORs (95% CI) of Low-WBC ^3^
Smoking (N, %)				
Non-smoker	3630 (87.0) ^1^	18,216 (76.4)	7700 (61.2) ^+++^	1
Former-smoker	412 (9.90)	3902 (16.4)	2236 (17.8)	0.352 (0.271–0.458)
Smoker	121 (2.91)	1723 (7.23)	2656 (21.1)	0.298 (0.230–0.387) ^###^
Regular exercise ^4^ (%)	2430 (58.3)	13,354 (56.0)	6453 (51.2) ^+++^	1.262 (1.165–1.367) ^###^
Alcohol intake ^5^ (≥20g/week)	1413 (33.8)	14,234 (42.5)	1498 (48.7) ***	0.849 (0.768–0.936) ^###^
Coffee ^6^ (cups/week)	3.5 (3.4–3.6) ^c^	3.7 (3.7–3.8) ^b^	4.0 (3.9–4.0) ^a,^***	0.856 (0.790–0.928) ^###^
Energy intake ^7^ (%EER)	94.7 (93.7–95.6) ^2,c^	96.2 (95.9–96.6) ^b^	95.4 (94.3–96.5) ^a,b,^**	0.949 (0.876–1.028)
CHO intake ^8^ (energy %)	71.7 (71.5–71.9) ^a^	71.7 (71.6–71.8) ^a^	71.4 (71.3–71.5) ^b,^*	0.983 (0.880–1.097)
Protein intake ^9^ (energy %)	13.4 (13.3–13.5)	13.4 (13.3–13.4)	13.4 (13.4–13.5)	0.993 (0.918–1.075)
Fat intake ^10^ (energy %)	13.9 (13.7–14.1) ^a^	13.9 (13.9–14.0) ^a^	14.1 (14.0–14.2) ^b,^**	0.952 (0.875–1.036)
Vitamin D ^11^ (ug/day)	6.48 (6.34–6.61) ^a^	6.39 (6.34–6.45) ^a^	6.23 (6.15–6.31) ^b,^**	1.080 (0.975–1.197)
Anti-inflammation index (scores) ^12^	1933 (1891–1975)	1926 (1908–1943)	1918 (1894–1943)	1.010 (0.917–1.113)
Korean balanced diet ^13^ (<66th per, N, %)	1176 (28.2)	7337 (30.7)	4245 (33.5) ^+++^	1.034 (0.893–1.198)
Plant-based diet ^13^ (N, %)	1625 (38.9)	8153 (34.1)	3611 (28.5) ^+++^	1.231 (1.041–1.456)
Western-style diet ^1^^3^ (N, %)	1181 (28.3)	7728 (32.3)	4676 (37.0) ^+++^	1.032 (0.818–1.303)
Rice-main diet ^13^ (N, %)	1417 (33.9)	7672 (32.1)	4205 (33.2) ^+^	1.079 (0.976–1.192)

^1^ Values represent the number (%) and ^2^ adjusted means ± standard deviations after adjusting for covariates or the number of the subjects and percentage. Covariates used were age, sex, body mass index (BMI), energy intake, income, education, residence area, survey year, having autoimmunity-related diseases including atopic dermatitis, asthma, allergy, having inflammation-related diseases including gastritis, alcohol intake, smoking, and physical activity. ^3^ Adjusted odds ratio (ORs) and 95% confidence intervals of each parameter for the hypo-WBC (<4.0 × 10^9^/L) risk the after adjusting for covariates using logistic regression analysis. The cutoff points were as follows: ^4^ < moderate intensity activity for 150 min/week; ^5^ <20 g alcohol/day, ^6^ <3 cups/week, ^7^ <estimated energy requirement (EER); ^8^ <65 carbohydrate (CHO) energy % (En%); ^9^ <15 protein En%; ^10^ <20 fat En%; ^11^ <9.4 ug V-D/day; ^12^ <2374 scores; ^13^ <66th percentiles. ^a,b,c^ Different letters indicate significant differences among the groups in the Tukey test at *p* < 0.05. * Significantly different for white blood cell (WBC) count groups by one-way ANCOVA in continuous variables at *p* < 0.05, ** at *p* < 0.01, and *** at *p* < 0.001. ^+^ Significantly different WBC count groups by χ^2^ test at *p* < 0.05, ^+++^ at *p* < 0.001. ^###^ Significant association of WBC count groups with each variable at *p* < 0.001.

**Table 3:** The characteristics of the ten genetic variants of genes related to immunity in the risk of low WBC count (<4.0 × 10^9^/L) and used for the generalized multifactor dimensionality reduction analysis.

**Chr.^1^**	**SNP ^2^**	**Position**	**Mi ^3^**	**Ma ^4^**	**OR ^5^** **(95% CI) ^6^**	***p*-Value Adjusted ^7^**	**OR ^8^** **(95% CI)**	***p*-Value Adjusted ^9^**	**MAF ^10^**	**HWE ^11^**	**Gene**	**Functional Consequence**
2	rs80157389	136546733	C	G	0.75 (0.69–0.81)	1.90 × 10^−13^	0.73 (0.59–0.904)	0.004	0.179	0.79	*LCT*	intron
6	rs2308575	31239057	T	C	0.82 (0.77–0.88)	4.90 × 10^−8^	0.78 (0.54–0.96)	0.029	0.202	0.349	*HLA-C*	missense
6	rs34791928	31781398	T	C	0.81 (0.72–0.92)	7.10 × 10^−4^	0.75 (0.50–0.97)	0.043	0.062	0.887	*HSPA1A*	near-gene-5
6	rs532162239	32558725	T	C	0.85 (0.80–0.90)	3.30 × 10^−8^	0.76 (0.59–0.93)	0.015	0.346	0.523	*HLA-DRB1*	upstream
6	rs112181319	33039694	T	G	0.86 (0.78–0.94)	9.50 × 10^−4^	0.73 (0.55–0.95)	0.012	0.107	0.546	*HLA-DPA1*	intron
6	rs3097649	33056962	T	C	1.10 (1.04–1.16)	9.00 × 10^−5^	1.16 (1.01–1.32)	0.043	0.363	0.95	*HLA-DPB1*	utr-3
6	rs3176337	36648920	A	C	0.86 (0.81–0.92)	4.90 × 10^−6^	0.77 (0.60–0.97)	0.035	0.245	0.697	*CDKN1A*	intron
7	rs445	92408370	T	C	1.18 (1.12–1.25)	8.61 × 10^−9^	1.21 (1.02–1.40)	0.002	0.327	0.888	*CDK6*	intron
17	rs9898547	38136026	T	G	1.23 (1.16–1.29)	2.40 × 10^−13^	1.45 (1.24–1.68)	2.2 × 10^−6^	0.399	0.475	*PSMD3*	near-gene-5
19	rs7502539	38219005	A	G	1.18 (1.12–1.25)	3.60 × 10^−9^	1.21 (1.03–1.42)	0.017	0.347	0.669	*THRA*	near-gene-5

^1^ Chromosome; ^2^ single nucleotide polymorphism; ^3^ minor allele; ^4^ major allele; ^5^ odds ratio (OR) for the hospital-based large cohort (case, *n* = 4176 and control, *n* = 36,551); ^6^ lower and upper end of 95% confidence interval (CI); ^7^ *p*-value for OR in the hospital-based large cohort after adjusting for age, gender, residence area, survey year, body mass index, daily energy intake, education, and income in logistic regression analysis; ^8^ OR for the Ansan/Ansung cohort; ^9^ *p*-value for OR in Ansan/Ansung cohort (case, *n* = 207; control, *n* = 5286); ^10^ minor allele frequency; ^11^ *p*-value for Hardy-Weinberg equilibrium.

**Table 4:** The characteristics of the ten genetic variants of genes in the risk of low white blood cell count applied for the generalized multifactor dimensionality reduction analysis (GMDR).

	Adjusted for Gender and Age				Adjusted for Gender, Age, Residence Area, BMI, and Serum CRP			
Model	TRBA	TEBA	*p*-Value	CVC	TRBA	TEBA	*p*-Value	CVC
***PSMD3***_rs9898547	0.5270	0.5247	10 (0.0010)	9/10	0.5270	0.5225	10 (0.0010)	6/10
**Model 1 plus** ***LCT*_rs80157389**	0.5391	0.5381	10 (0.0010)	10/10	0.5392	0.5383	10 (0.0010)	10/10
Model 2 plus ***HLA-C***_rs2308575	0.5421	0.5334	10 (0.0010)	5/10	0.5425	0.5328	10 (0.0010)	4/10
Model 2 plus ***HLA-DRB1*** _rs532162239 ***HLA_DPB1*** _rs3097649	0.5486	0.5351	10 (0.0010)	7/10	0.5494	0.5349	10 (0.0010)	8/10
Model 4 plus ***CDKN1A***_rs3176337	0.5589	0.5273	10 (0.0010)	6/10	0.5597	0.5304	10 (0.0010)	7/10
Model 5 plus ***HLA-C***_rs2308575	0.5758	0.5177	10 (0.0010)	5/10	0.5768	0.5208	10 (0.0010)	5/10
**Model 6 plus** *****THRA***_rs7502539**	0.6028	0.5259	10 (0.0010)	10/10	0.6040	0.5291	10 (0.0010)	10/10
**Model 7 plus** ***HLA-DPA1*_rs112181319**	0.6254	0.5239	10 (0.0010)	10/10	0.6260	0.5248	10 (0.0010)	10/10
**Model 8 plus** ***HSPA1A*_rs34791928**	0.6447	0.5189	9 (0.0107)	10/10	0.6452	0.5215	10 (0.0010)	10/10
**Model 9 plus** ***CDK6*_rs445**	0.6559	0.5198	10 (0.0010)	10/10	0.6561	0.5218	10 (0.0010)	10/10

TRBA, trained balanced accuracy; TEBA, test balance accuracy; CVC, cross-validation consistency; sign test, result, and *p*-value for the significance of GMDR model by sign test with and without adjusting for covariates designated; BMI, body mass index. Bold faces indicated the best models.

**Table 5:** Adjusted odds ratios of polygenetic risk scores of the best model (PRS) for the hypo-WBC risk after covariate adjustments according to lifestyles patterns and the interaction of PRS with lifestyles for the hypo-WBC risk.

	Low-PRS (*n* = 2719)	Medium-PRS (*n* = 11150)	High-PRS (*n* = 26,899)	Gene-Nutrient Interaction *p*-Value
Low energy ^1^ High energy	1	1.401(1.074–1.829) 1.625(1.158–2.280)	2.130(1.657–2.739) 2.104(1.522–2.909)	0.3184
Low CHO ^2^ High CHO	1	2.020 (1.132–3.606) 1.412 (1.128–1.767)	2.659 (1.525–4.635) 2.038 (1.648–2.521)	0.3799
Low protein ^3^ High protein	1	1.316(0.992–1.745) 1.547(1.276–1.875)	1.879(1.439–2.453) 1.718(1.256–2.349)	0.6677
Low fat ^4^ High fat	1	1.656 (1.165–1.819) 1.227(0.939–2.177)	2.085 (1.688–2.575) 2.638(1.307–4.184)	0.0170
Low KBD ^5^ High KBD	1	1.325 (1.044–1.682) 1.603 (1.232–2.086)	1.928 (1.539–2.415) 2.285 (1.780–2.935)	0.2819
Low PBD ^5^ High PBD	1	1.266 (0.979–1.638) 1.454 (1.171–1.805)	1.924 (1.510–2.451) 2.089 (1.702–2.564)	0.2670
Low WSD ^5^ High WSD	1	1.434 (1.117–1.839) 1.428 (1.114–1.829)	2.118 (1.673–2.681) 1.937 (1.531–2.449)	0.1327
Low RMD ^5^ High RMD	1	1.533 (1.175–2.001) 1.468 (1.156–1.917)	2.210 (1.716–2.846) 2.126 (1.672–2.703)	0.4678
Low exercise ^6^ High exercise	1	1.287 (0.949–1.746) 1.651 (1.238–2.202)	1.799 (1.349–2.398) 2.371 (1.803–3.120)	0.0482

According to the low and high intake groups, values represent adjusted odds ratio (OR) and 95% confidence intervals. Covariates were age, sex, body mass index (BMI), energy intake, income, education, residence area, survey year, taking immune-related medicine, alcohol intake, smoking status, and physical activity. The cutoff points were as follows: ^1^ <estimated energy requirement (EER); ^2^ <65 carbohydrate (CHO) energy % (En%); ^3^ <15 protein En%; ^4^ <20 fat En%; ^5^ <66th percentiles; ^6^ <moderate-intensity exercise for 150 min/week. KBD, Korean balanced diet; PBD, plant-based diet; WSD, Western-style diet, and RMD, rice-main diet. PRS was divided into three categories (0–4, 5–6, and ≥7) as the low-PRS, medium-PRS, and high-PRS groups of the best model of GMDR with 5 SNPs. Logistic regression models include the corresponding main effects, interaction terms of gene and main effects, and covariates. Reference was the low-PRS.

## Data Availability

The authors’ raw data involved in this study will be available to any qualified researcher.

## Supplementary Materials

The following are available online at https://www.mdpi.com/article/10.3390/nu13082849/s1, Table S1. Factor loadings of food groups in dietary patterns identified using principal component analysis Table S2. The characteristics of the 19 genetic variants of genes related to immunity in the risk of low WBC count. Table S3. After covariate adjustments, they adjusted OR of metabolic syndrome risk with polygenetic risk scores of the 7 SNPs model (PRS) for gene–gene interaction.

## Abbreviations

MetS: metabolic syndrome; WBC, white blood cells; CRP, serum C-reactive protein concentrations; KoGES, Korean Genome and Epidemiology Study; SQFFQ, semi-quantitative food frequency questionnaire; ORs, odds ratios; CI, 95% confidence intervals; PRS, polygenetic risk scores; HLA, human leukocyte antigen; IL, interleukin; CD4, cluster of differentiation 4; BMI, Body mass index; DII, Dietary inflammatory index; GMDR, generalized multifactor dimensionality reduction; TRBA, trained balanced accuracy; TEBA, testing balanced accuracy; PSMD3, proteasome 26S Subunit, non-ATPase 3; LCT, lactase; THRA, thyroid hormone receptor alpha; CDKN1A, cyclin-dependent kinase inhibitor 1A.
