# Supplementary material for: Association between Polygenetic Risk Scores of Low Immunity and Interactions between These Scores and Moderate Fat Intake in a Large Cohort
Source: Nutrients. 2021 Aug 19;13(8):2849. doi: 10.3390/nu13082849 (PMC8402209; doi:10.3390/nu13082849)
Supplement: Supplementary file 1 [file nutrients-13-02849-s001.zip › nutrients-1317932-supplementary.pdf]

Table S1. Factor loadings of food groups in dietary patterns identified using principle component analysis

	Factor 1		Factor 2		Factor 3		Factor 4	
Rice	-2		-7		3		93	*
Grain	8		-5		-2		-93	*
Noodles	3		1		62	*	3	
Breads	-5		35		53	*	-4	
Cookies	-4		29		31		6	
Beans	36		44	*	3		1	
Potatoes	27		49	*	6		-2	
Kimchi	51	*	-3		-1		-3	
Eggs	11		41	*	16		5	
Fast foods	-3		15		76	*	-3	
Green vegetables	69	*	39		-2		-2	
Mushroom	72	*	25		1		1	
White vegetables	51	*	33		-5		-4	
Fatty fish	54	*	20		11		0	
White fish	66	*	14		12		0	
Crabs	49	*	2		18		1	
Processed meats	20		13		6		-2	
Red meats	46	*	-11		40		8	
Soups	16		2		66	*	-4	
Chickens	33		-8		38		4	
Seaweeds	46	*	38		-2		-4	
Milk	14		48	*	2		1	
Beverages	22		29		6		2	
Coffee	11		-19		16		13	
Tea	14		-11		23		14	
Fruit	22		46	*	-5		-5	
Pickle	50	*	-4		4		1	

Alcohol	17	-30	15	5
Nuts	1	50 *	7	-4
Variance Explained by Each Factor	3.710	2.328	2.289	1.797

Printed values are multiplied by 100 and rounded to the nearest integer. Values greater than 0.4 are flagged by an '*'.

Supplemental Table S2. The characteristics of the 19 genetic variants of genes related to immunity in the risk of low WBC count

Chr.[1]	SNP	Position	Mi[2]	Ma[3]	OR[4] (95% CI)[5]	P-value adjusted[6]	MAF[7]	HWE[8]	Gene	Functional Consequence
2	rs80157389	136546733	C	G	0.75 (0.69-0.81)	1.90E-13	0.179	0.79	*LCT*	intron
6	rs2308575	31239057	T	C	0.82 (0.77-0.88)	4.90E-08	0.202	0.349	*HLA-C*	missense
6	rs34791928	31781398	T	C	0.81 (0.72-0.92)	7.10E-04	0.062	0.887	*HSPA1A*	near-gene-5
6	rs532162239	32558725	T	C	0.85 (0.80-0.90)	3.30E-08	0.346	0.523	*HLA-DRB1*	upstream
6	rs112181319	33039694	T	G	0.86 (0.78-0.94)	9.50E-04	0.107	0.546	*HLA-DPA1*	intron
6	rs3097649	33056962	T	C	1.1 (1.04-1.16)	9.00E-05	0.363	0.95	*HLA-DPB1*	utr-3
6	rs3176337	36648920	A	C	0.86 (0.81-0.92)	4.90E-06	0.245	0.697	*CDKN1A*	intron
7	rs445	92408370	T	C	1.18 (1.12-1.25)	8.61E-09	0.327	0.888	*CDK6*	Intron
17	rs9898547	38136026	T	G	1.23 (1.16-1.29)	2.40E-13	0.399	0.475	*PSMD3*	near-gene-5
19	rs7502539	38219005	A	G	1.18 (1.12-1.25)	3.60E-09	0.347	0.669	*THRA*	near-gene-5
6	rs566510875	2065566	A	G	1.38 (1.18-1.62)	6.6.E-05	0.02311	0.1691	*GMDS*	intron
6	rs1264455	30462381	A	G	1.10 (1.04-1.16)	8.9.E-04	0.3673	0.342	*HLA-E*	intron
7	rs445	92408370	T	C	1.18 (1.12-1.25)	8.6.E-09	0.3273	0.8882	*CDK6*	intron
8	rs76971655	18487885	T	C	1.32 (1.14-1.53)	1.8.E-04	0.02894	0.1223	*PSD3*	intron
11	rs2729763	77031672	G	A	1.12 (1.06-1.19)	3.2.E-04	0.2287	0.4611	*PAK1*	near-gene-3
15	rs74871911	81508611	T	C	1.12 (1.05-1.20)	6.9.E-04	0.1945	0.3992	*IL16*	intron
16	rs869160384	68771418	C	T	1.11 (1.04-1.18)	8.3.E-04	0.2725	0.1479	*CDH1*	intron
16	rs12935142	78533032	T	C	1.16 (1.08-1.24)	7.7.E-05	0.157	0.3997	*WWOX*	intron
17	rs117214949	38184283	T	C	1.20 (1.12-1.28)	2.4.E-07	0.1718	0.7607	*MED24*	intron

[1]Chromosome; [2]Single nucleotide polymorphism; [3]Minor allele; [4]Odds ratio; [5]Lower and Upper ends of 95% confidence interval (CI); [6] P-value for OR after adjusting for age, gender, residence area, survey year, body mass index, daily energy intake, education, and income; [7]Minor allele frequency; [8] P-value for Hardy-Weinberg equilibrium.

Table S3. Adjusted odds ratios (OR) for the risk of metabolic syndrome and its components by polygenetic risk scores of the 7 SNPs model (PRS) for gene-gene interaction after covariate adjustments

	Model 1			Model 2	
	Low-PRS[1] (n=2,719)	Medium-PRS (n=11,150)	High-PRS (n=26,899)	Medium-PRS (n=11,150)	High-PRS (n=26,899)
Cancer (Yes)	1	1.148 (0.914-1.443)[2]	1.122 (0.904-1.393	1.114 (0.865-1.434)	1.103 (0.868-1.401)
MetS (Yes)	1	1.000 (0.880-1.137)	0.957 (0.848-1.079)	1.014 (0.866-1.188)	0.931 (0.803-1.081)
BMI (≥25 kg/m^2)	1	1.000 (0.910-1.099)	1.027 (0.940-1.122)	1.034 (0.927-1.154)	1.069 (0.964-1.185)
Body fat[3] (%)	1	0.972 (0.886-1.066)	1.003 (0.920-1.094)	0.989 (0.888-1.100)	1.026 (0.928-1.135)
Waist [4] (cm)		0.917 (0.822 1.024)	0.949 (0.856-1.051)	0.983 (0.865-1.118)	1.014 (0.899-1.144)
Type 2 diabetes[5]	1	0.955 (0.824 1.108)	0.932 (0.811-1.071)	0.909 (0.772-1.071)	0.890 (0.763-1.038)
Blood pressure[6]	1	1.004 (0.901 1.119)	0.986 (0.891-1.092)	1.036 (0.914-1.174)	1.036 (0.914-1.174)
LDL-C[7] (mg/dl)	1	0.961 (0.838-1.103)	0.952 (0.837-1.084)	0.943 (0.823-1.082)	0.924 (0.812-1.052)
HDL-C[8] (mg/dl)	1	1.003 (0.893-1.127)	1.013 (0.908-1.130)	0.991 (0.883-1.112)	0.985 (0.883-1.098)
Triglyceride[9]	1	1.066 (0.951-1.195)	1.046 (0.939 1.165	1.074 (0.958-1.204)	1.051 (0.944 1.171
Hs-CRP[10]	1	0.989 (0.769-1.271)	1.031 (0.815 1.305)	0.978 (0.761-1.258)	1.029 (0.813-1.302)

[1]PRS of 7-SNPs in the best model were calculated by summing the number of risk alleles of SNPs. The PRS was divided into three categories (0-5, 6-7, and ≥8).

[2]Adjusted OR and 95% confidence intervals (CI) after adjusting the covariates including age, sex, body mass index (BMI), energy intake, income, education, residence area, survey year,

white blood cell counts, autoimmunity-related diseases, including atopic dermatitis, asthma, allergy, and inflammation-related diseases, alcohol intake, smoking status, and physical activity.

The cutoff points were as following: [3]≥ 25% fat mass for men and 30% for women for fat mass; [4]≥90 cm waist circumferences for men ≥85 cm for women; [5] ≥126 mg/dL fasting serum glucose or ≥6.5% HbA1c or taking hypoglycemic medication; [6]≥130 mmHg SBP and ≥90 mmHg DBP or taking hypotensive medication; [7]≥160 mg/dl serum LDL; [8]≤40 mg/dL for men and ≤50 mg/dL serum HDL; [9] ≥150 mg/dl serum triglyceride. MetS, metabolic syndrome; [7] ≥ 0.5 mg/dL serum high-sensitive C-reactive protein (CRP).

No statistical significance in ORs of each variable at $P<0.05$.
